# New Test for Bile and Sugar

**Published:** 1846-05-01

**Authors:** 


*New Test for Bile and Sugar.*—This test is based upon the deep violet tint afforded by the addition of sulphuric acid and sugar to the bile even when perfectly colorless. It is upon the choleic acid (which forms the essential part of the bile) that this reaction takes place. A little of the liquid suspected to contain the bile is poured into a test tube, and two-thirds of sulphuric acid added by drops, so as not to allow the temperature to exceed 144° Fahr., or a higher temperature than would decompose the choleic acid; then add from two to five drops of a solution of one part of sugar to four-fifths of water, and shake the mixture: if bile be present, the violet red colour will appear in a shorter or longer space of time according to the quantity present. The precautions necessary to succeed are, not to allow the temperature to exceed 144° ; not to add too much sugar; the sulphuric acid must be free from sulphurous acid. If albumen be present in the suspected liquor, it is best to coagulate it previous to testing, with a little alcohol or heat. If the bile be in a small quantity, it should be concentrated in a water bath, extracted with alcohol, and this test evaporated to a *small* bulk, and the test applied to the solution when cold; this is particularly to be attended to, when the urine and other secretions are the subjects of experiment. By means of this test bile was detected in the urine of a patient with pneumonia. The fœces of a healthy man, when extracted with spirits, and tested, did not show any indications of bile, whereas in adding a little bile previously to the fœces, the test did not fail to indicate it. This test reversed may be used for the detection of sugar, that is to say, a mixture of bile and sulphuric acid is first made, and the suspected liquid added; if sugar be present, the violet red color will appear. This is a ready way for testing diabetic urine.

To test the blood for bile, the albumen is first separated by boiling with alcohol, and the concentrated solution tested as already mentioned.

Dr. M. Pettenkoffer—(Ann. der Chem. and Pharm.)—*From American Jorrnal of Sciecne and Arts.*

